# Residual power series scheme treatments for fractional Klein-Gordon problem arising in soliton theory

**DOI:** 10.1038/s41598-024-79247-9

**Published:** 2024-12-23

**Authors:** Saad Z. Rida, Anas A. M. Arafa, Hussein S. Hussein, Ismail Gad Ameen, Marwa M. M. Mostafa

**Affiliations:** 1 Department of Mathematics, Faculty of Science, South Valley University, Qena, 83523 Egypt; 2 Department of Mathematics, College of Science, Qassim University, Buraydah, Saudi Arabia

**Keywords:** Confluent Bernoulli polynomials, Residual power series scheme, Fractional derivatives, Klien-Gordon equations, Numerical results, Mathematics and computing, Computational science, Pure mathematics

## Abstract

The Klein-Gordon problem (KGP) is one of the interesting models that appear in many scientific phenomena. These models are characterized by memory effects, which provide insight into complex phenomena in the fields of physics. In this regard, we propose a new robust algorithm called the confluent Bernoulli approach with residual power series scheme (CBCA-RPSS) to give an approximate solution for the fractional nonlinear KGP. The convergence, uniqueness and error analysis of the proposed method are discussed in detail. A comparison of the numerical results obtained by CBCA-RPSS with the results obtained by some well-known algorithms is presented. Numerical simulations using base errors indicate that CBCA-RPSS is an accurate and efficient technique and thus can be used to solve linear and nonlinear fractional models in physics and engineering. All the numerical results for the studied problems were obtained through implementation codes in Matlab R2017b.

## Introduction

Fractional calculus (FC) is a powerful and effective tool for solving many differential equations. It is a branch of mathematical analysis that deals with the generalization of fractional integrals and derivatives. Over the past few decades, FC has attracted much interest due to its applications in fields of sciences. Many phenomena in various science are modeled by fractional partial differential equations (FPDEs) for example mathematical physics, plasma physics, optics, engineering, quantum evolution of complex systems, chemical physics, fractional dynamics, biology, electromagnetic waves, astrophysics, wave propagation phenomenon, and image processing^1–8^.

Many researchers have been interested in solving FPDEs by many effective and explicit methods. Researchers have presented many numerical and analytical methods to study these equations such as Adomian decomposition Laplace transform method (ADLTM)^9,10^, finite element method^11^, finite Hankel transform procedure^12^, collocation method^13^, Adomian decomposition general transform method^14^, Q-homotopy analysis transform method^15^, Sawi transform with the homotopy perturbation method^16^, modified auxiliary equation method^17^, Shehu transform and ADM^18^, homotopy perturbation general transform method^19^, $exp(\Phi (\zeta ))$-expansion method^20^ and Adomian decomposition formable transform method^21^.

Among these techniques, the RPSS is characterized by simplicity and power as it was first introduced by El-Ajou to find the solution of fuzzy differential equations of the first and second order^22^. RPSS does not require comparison of the coefficients of the corresponding terms and the recurrence relation. It can be used directly for the target problem by choosing a suitable initial guess approximation. The solution of RPSS is based on obtaining power series for linear and nonlinear problems without discretization, perturbation or linearization^22,23^. Over the past few years, RPSS has been applied to solve many ordinary and linear and nonlinear partial differential equations^24–30^.

KGP is one of the important mathematical physics models that describe dispersive wave phenomena. In addition, it has many applications such as nonlinear wave propagation, plasma physics, nonlinear optics, fluid dynamics, quantum field theory, relativistic physics, dispersive wave phenomena, condensed matter physics, chemical kinetics and solid-state physics^31–36^. The nonlinear fractional time KGP is defined as follows^37^:

1 $$\begin{aligned} \mathcal {D}^{\zeta }_{\tau }\ \Psi ({z},\tau )+\rho \ \mathcal {D}^{2}_{ z }\ \Psi ({z},\tau )+\sigma \ \Psi ({z},\tau )+\varrho \ \Psi ^{2}({z},\tau )={\mathbb {H}}({z},\tau ), \ 1<\zeta \le 2,\ {z}\in [-1,1],\ \tau >0, \end{aligned}$$

with to initial conditions (ICs) and boundary conditions (BCs):

2 $$\begin{aligned} {\left\{ \begin{array}{ll} \Psi ({z},0)=\mathfrak {F}_1 ({z}), & \mathcal {D}_{\tau } \Psi ({z},0)=\mathfrak {F}_2 ({z}),\\ \Psi (-1,\tau )=-\mathcal {W} (\tau ), & \Psi (1,\tau )=\mathcal {W}(\tau ), \end{array}\right. } \end{aligned}$$

where $\Psi ( z ,\tau )$ is wave displacement at $z$ and $\tau$, $\mathfrak {F}_1 ( z )$ and $\mathfrak {F}_1 ( z )$ are wave kinks, $\mathbb {H}( z ,\tau )$ is the source function and $\rho ,\ \sigma ,\ \varrho \ \in \textbf{R}$.

Several numerical and analytical techniques are discussed for solving KGP. For instance, Haar wavelets method^38^, spline collocation method^39^, natural homotopy perturbation method^40,41^, Cubic trigonometric B-spline basis functions^37^, Tension spline approach^42^, radial basis functions (RBF)^43^, clique polynomial method^44^, Bernoulli wavelet collocation method (BWCM)^45^, Sinc Chebyshev collocation method^46^, Clique polynomials^47^, variational iteration method^48^, Sobolev Gradien^49^, $\bigg (\dfrac{G'}{G}\bigg )$ expansion method^50^ and homotopy analysis transform method^51^.

The primary aim of this paper is to present semi analytical technique in order to find the solution for FPDEs in general that is considered new accurate technique and can be applied to obtain the solution for many physical and engineering applications containing FPDEs in a few straightforward steps. Moreover, we utilized CBCA-RPSS for solving KGP which defined in Eqs. (1) and (2). Hence, we compared error norms obtained by CBCA-RPSS with other existing techniques in the literature. As far as we know, CBCA-RPSS has never been used to solve KGP, which is what motivated us to carry out this study.

This current study is designed in the following manner: In “Essential definitions” section, we introduced some mathematical definitions which are utilized in this paper. Main results are presented in “Main results” section. In “Description of methodology” section, we explained the CBCA-RPSS for solving non-linear time-fractional KGP. The proposed problems and norm errors are presented in “The proposed problems and norm errors” section. To illustrate the efficiency and accuracy for CBCA-RPSS, the numerical results and discussion are given in “Results and discussion” section. Finally, “Concluding remarks” section is provided concluding remarks.

## Essential definitions

In this section, we present Caputo’s fractional partial derivative (CFPD) and some of its major features. Also essential concepts of fractional power series (FPS) and Bernoulli polynomials are revisited.

### Definition 1

The CFPD $\mathcal {D}^{\zeta }$ of order $\zeta$ for the given function $\mathcal {U}( z ,\tau )$ is defined as^5,52^:

3 $$\begin{aligned} \mathcal {D}^{\zeta }_{\tau }\ \mathcal {U}({z},\tau )= \left\{ \begin{array}{ll} \frac{1}{\Gamma (n-\zeta )}\displaystyle \int _{0}^{\tau } (\tau -\upsilon )^{n-\zeta -1}\ \frac{\partial ^{n}\mathcal {U}({z},\upsilon )}{\partial \upsilon ^{n}} d\upsilon ,\quad n-1<\zeta < n,\\ \frac{\partial ^{n} \mathcal {U}({z},\tau )}{\partial \tau ^{n}}, \qquad \zeta =n\in \mathbb {N}. \\ \end{array} \right. \end{aligned}$$

The CFD satisfies linear property similar to integer order differentiation:

$$\begin{aligned} \mathcal {D}^\zeta \bigg (C_1 \mathfrak {A}_{1}(\tau )+C_2 \mathfrak {A}_{2}(\tau )\bigg )=\bigg (C_1 \mathcal {D}^\zeta \mathfrak {A}_{1}(\tau )+C_2 \mathcal {D}^\zeta \mathfrak {A}_{2}(\tau ) \bigg ), \end{aligned}$$

where $C_1, C_2$ are constants. For CFD, we obtain the major properties as below:

4 $$\begin{aligned} \mathcal {D}^{\zeta } \mathbb {K}= & 0, \ \mathbb {K} \quad is ~ constant. \end{aligned}$$

5 $$\begin{aligned} \mathcal {D}^{\zeta } \tau ^{\omega }= & \left\{ \begin{array}{ll}\frac{\Gamma (\omega +1)}{\Gamma (\omega +1-\zeta )}\tau ^{\omega -\zeta }, & \text { for } \omega \in \mathcal {N}_{0},~ \omega \ge \lceil \zeta \rceil ,\\ 0, & \text { for } \omega \in \mathcal {N}_{0},~ \omega < \lceil \zeta \rceil , \\ \end{array}\right. \end{aligned}$$

where $\lceil \zeta \rceil$ denote to the smallest integer greater than or equal to $\zeta$, where $\mathcal {N}_{0}= \{0,1,2,...\}$.

### Definition 2

The *sl*-truncated series $v_{s l}( z ,\tau )$ of the RPSS is given as below^24,53^:

6 $$\begin{aligned} v_{s l}( z ,\tau )= \sum _{r=0}^{m-1} \dfrac{\mu _{r}( z )}{r!} +\sum _{r=1}^s\sum _{j=0}^l \vartheta _{rj}( z )\dfrac{\tau ^{r\eta +j}}{\Gamma (r\eta +j+1)},\tau >0,\ r-1<\eta \le r, \end{aligned}$$

and

7 $$\begin{aligned} v_{s l}( z ,\tau )= \sum _{r=0}^{m-1} \dfrac{\Omega _{r}(\tau )}{r!} +\sum _{r=1}^s\sum _{j=0}^l P_{rj}(\tau )\dfrac{ z ^{r\kappa +j}}{\Gamma (r\kappa +j+1)}, z >0,\ r-1<\kappa \le r. \end{aligned}$$

### Definition 3

Bernoulli orthonormal polynomial of order *n* can be defined in^54^ as:

8 $$\begin{aligned} \mathfrak {B}_n( z )=\sqrt{2n+1}\sum _{i=0}^n\ (-1)^i \left( {\begin{array}{c}n\\ i\end{array}}\right) \left( {\begin{array}{c}2n-i\\ n-i\end{array}}\right) z ^{n-i},\quad n=0,1,2,.... \end{aligned}$$

According to the orthogonal property, we obtain:

$$\begin{aligned} <\mathfrak {B}_n( z ),\mathfrak {B}_m( z )>=\displaystyle \int _{0}^{1} \mathfrak {B}_n( z )\mathfrak {B}_m( z ) dz={\left\{ \begin{array}{ll} 0, & \hbox { if}\ n\ne m,\\ 1, & \text {if } n= m.\\ \end{array}\right. } \end{aligned}$$

### Definition 4

The confluent Bernoulli polynomials is described as below^55^:

9 $$\begin{aligned} \mathfrak {B}^{(a,b)}_n( z )=\sum _{i=0}^n\ \left( {\begin{array}{c}n\\ i\end{array}}\right) \frac{\ (a)_{n-i}}{(b)_{n-i}}\ \mathbb {B}_i\ z ^{n-i},\ \end{aligned}$$

where $\mathbb {B}_i$ is Bernoulli numbers are known that $\mathbb {B}_0=1, \ \mathbb {B}_1=\dfrac{-1}{2},\ \mathbb {B}_2=\dfrac{1}{6};\ \mathbb {B}_{2i+1}=0\ (i=1,2,...)$.

The function $\Psi ( z ,\tau )$ is defined in terms of confluent Bernoulli polynomials as:

10 $$\begin{aligned} \Psi ( z ,\tau )=\sum _{i=0}^\infty \lambda _{i}(\tau )\ \mathfrak {B}^{(a,b)}_i( z ). \end{aligned}$$

In practice, we truncate the infinite series up to $(n+1)$ terms of confluent Bernoulli polynomials as follows:

11 $$\begin{aligned} \Psi _{n}( z ,\tau ) =\sum _{i=0}^n \lambda _{i}(\tau )\ \mathfrak {B}^{(a,b)}_i( z ). \end{aligned}$$

## Main results

Here, we conclude an approximate formula for CFD of function $\Psi _n( z ,\tau )$, convergence, uniqueness and error analysis as following theories:

### ** Fractional derivative for confluent Bernoulli polynomials**

#### Theorem 1

Assume that $\Psi _{n}( z ,\tau )$ be series approximation of confluent Bernoulli which defined by Eq. (11), then $\mathcal {D}^{\zeta } _{ z } \ \Psi _{n}( z ,\tau )$ is given as:

12 $$\begin{aligned} \mathcal {D}^{\zeta } _{ z } \ \Psi _{n}( z ,\tau )=\sum _{i=\lceil \zeta \rceil }^n \sum _{k=0}^{i-\lceil \zeta \rceil } \lambda _{i}(\tau )\ \mathcal {E}_{i,k} ^{(\zeta )}\ z ^{i-k-\zeta }, \end{aligned}$$

where $\mathcal {E}_{i,k} ^{(\zeta )}$ is defined as:

$$\begin{aligned} \mathcal {E}_{i,k} ^{(\zeta )}= \left( {\begin{array}{c}i\\ k\end{array}}\right) \frac{\ (a)_{i-k}\ \varGamma (i-k+1)}{ (b)_{i-k}\ \varGamma (i-k-\zeta +1)}\ \mathbb {B}_k. \end{aligned}$$

Proof: From the linear properties of CFD, we get

13 $$\begin{aligned} \mathcal {D}^{\zeta } _{ z } \ \Psi _{n}( z ,\tau )=\sum _{i=0}^n \lambda _{i}(\tau )\ \mathcal {D}^{\zeta }_{ z }\bigg (\mathfrak {B}^{(a,b)}_i( z )\bigg ). \end{aligned}$$

By appling Eqs. (4) and (5), we have $\mathcal {D}^{\zeta } _{ z }\bigg (\mathfrak {B}^{(a,b)}_i( z )\bigg )=0,\quad i=0,1,2,... \lceil \zeta \rceil -1,\quad \zeta >0$.

Also, $\forall \ i=\lceil \zeta \rceil , \lceil \zeta \rceil +1,...,n$, we get

14 $$\begin{aligned} \mathcal {D}^{\zeta } _{ z }\mathfrak {B}^{(a,b)}_i( z )=\sum _{k=0}^i \left( {\begin{array}{c}i\\ k\end{array}}\right) \frac{(a)_{i-k}}{ (b)_{i-k}}\ \mathbb {B}_k \mathcal {D}^{\zeta } _{ z } \bigg ( z ^{i-k}\bigg )\\= \sum _{k=0}^{i-\lceil \zeta \rceil }\left( {\begin{array}{c}i\\ k\end{array}}\right) \frac{ (a)_{i-k}\ \varGamma (i-k+1)}{(b)_{i-k}\ \varGamma (i-k-\zeta +1)}\ \mathbb {B}_k \ z ^{i-k-\zeta }, \end{aligned}$$

by substituting from Eq. (14) in Eq. (13), we obtain

15 $$\begin{aligned} \mathcal {D}^{\zeta } _{ z } \ \Psi _{n}( z ,\tau )=\sum _{i=\lceil \zeta \rceil }^n \sum _{k=0}^{i-\lceil \zeta \rceil } \lambda _{i}(\tau )\ \mathcal {E}_{i,k} ^{(\zeta )}\ z ^{i-k-\zeta },\nonumber \\ \mathcal {E}_{i,k} ^{(\zeta )}= \left( {\begin{array}{c}i\\ k\end{array}}\right) \frac{(a)_{i-k}\ \varGamma (i-k+1)}{ (b)_{i-k}\ \varGamma (i-k-\zeta +1)}\ \mathbb {B}_k. \end{aligned}$$

### **Convergence, uniqueness and error analysis **

#### Theorem 2

Let $\textbf{H}$ be a Hilbert space. Then the series solution $\displaystyle \sum _{i=0}^\infty \lambda _{i}(\tau )\ \mathfrak {B}^{(a,b)}_i( z )$ of Eqs. (1) and (2) converges if there exist $\Delta >0$, such that    $\Arrowvert \lambda _{i+1}(\tau )\ \mathfrak {B}^{(a,b)}_{i+1}( z )\Arrowvert \le \Delta \Arrowvert \lambda _{i}(\tau )\ \mathfrak {B}^{(a,b)}_{i}( z )\Arrowvert$.

#### Proof

We define sequence of partial sums as: $\mathbb {V}_{0}= \lambda _{0}(\tau )\ \mathfrak {B}^{(a,b)}_0( z )$,

$$\mathbb {V}_{1}= \lambda _{0}(\tau )\ \mathfrak {B}^{(a,b)}_0( z )+\lambda _{1}(\tau )\ \mathfrak {B}^{(a,b)}_1( z ),$$

$$\mathbb {V}_{2}= \lambda _{0}(\tau )\ \mathfrak {B}^{(a,b)}_0( z )+\lambda _{1}(\tau )\ \mathfrak {B}^{(a,b)}_1( z )+\lambda _{2}(\tau )\ \mathfrak {B}^{(a,b)}_2( z ),$$

. . .

$$\mathbb {V}_{i}= \lambda _{0}(\tau )\ \mathfrak {B}^{(a,b)}_0({z})+\lambda _{1}(\tau )\ \mathfrak {B}^{(a,b)}_1( z )+\lambda _{2}(\tau )\ \mathfrak {B}^{(a,b)}_2({z})+...+\lambda _{i}(\tau )\ \mathfrak {B}^{(a,b)}_i({z}).$$

We need to prove that $\{\mathbb {V}_{i}\}_{i=0}^{\infty }$ is convergent Cauchy sequence. Moreover, we consider

$$\Arrowvert \mathbb {V}_{i+1}- \mathbb {V}_{i}\Arrowvert =\Arrowvert \lambda _{i+1}(\tau )\ \mathfrak {B}^{(a,b)}_{i+1}({z})\Arrowvert \le \Delta \Arrowvert \lambda _{i}(\tau )\ \mathfrak {B}^{(a,b)}_{i}({z})\Arrowvert \le \Delta ^2\Arrowvert \lambda _{i-1}(\tau )\ \mathfrak {B}^{(a,b)}_{i-1}({z})\Arrowvert ...\le \Delta ^{i+1}\Arrowvert \lambda _{0}(\tau )\ \mathfrak {B}^{(a,b)}_{0}({z})\Arrowvert$$.

For $i,\mu \in \mathcal {N},i> \mu$, it yields

$$\begin{aligned}\Arrowvert \mathbb {V}_{i}- \mathbb {V}_{\mu }\Arrowvert =\Arrowvert \mathbb {V}_{i}- \mathbb {V}_{i-1}+\mathbb {V}_{i-1}-\mathbb {V}_{i-2}+...+\mathbb {V}_{\mu +1}-\mathbb {V}_{\mu }\Arrowvert . \end{aligned}$$

By using triangle inequality, we get

$$\Arrowvert \mathbb {V}_{i}- \mathbb {V}_{i-1}+\mathbb {V}_{i-1}-\mathbb {V}_{i-2}+...+\mathbb {V}_{\mu +1}-\mathbb {V}_{\mu }\Arrowvert \le \Arrowvert \mathbb {V}_{i}- \mathbb {V}_{i-1}\Arrowvert +\Arrowvert \mathbb {V}_{i-1}- \mathbb {V}_{i-2}\Arrowvert +...+\Arrowvert \mathbb {V}_{\mu +1}- \mathbb {V}_{\mu }\Arrowvert .$$

Further, we obtain

$$\begin{aligned} \Arrowvert \mathbb {V}_{i}- \mathbb {V}_{\mu }\Arrowvert \le \Arrowvert \mathbb {V}_{i}- \mathbb {V}_{i-1}\Arrowvert +\Arrowvert \mathbb {V}_{i-1}- \mathbb {V}_{i-2}\Arrowvert +...+\Arrowvert \mathbb {V}_{\mu +1}- \mathbb {V}_{\mu }\Arrowvert \\ \le \Delta ^{i}\Arrowvert \lambda _{0}(\tau )\ \mathfrak {B}^{(a,b)}_{0}( z )\Arrowvert +\Delta ^{i-1} \Arrowvert \lambda _{0}(\tau )\ \mathfrak {B}^{(a,b)}_{0}( z )\Arrowvert \\ +\Delta ^{i-2} \Arrowvert \lambda _{0}(\tau )\ \mathfrak {B}^{(a,b)}_{0}( z )\Arrowvert +...+\Delta ^{\mu +1}\Arrowvert \lambda _{0}(\tau )\ \mathfrak {B}^{(a,b)}_{0}( z )\Arrowvert \\ = \Arrowvert \lambda _{0}(\tau )\ \mathfrak {B}^{(a,b)}_{0}( z )\Arrowvert \bigg (\Delta ^{i}+\Delta ^{i-1}+\Delta ^{i-2}+...+\Delta ^{\mu +1}\bigg )\\ \le \dfrac{1-\Delta ^{i-\mu }}{1-\Delta } \Delta ^{\mu +1}\Arrowvert \lambda _{0}(\tau )\ \mathfrak {B}^{(a,b)}_{0}( z )\Arrowvert \rightarrow 0 \quad as \quad i, \mu \rightarrow \infty . \end{aligned}$$

Hence, $\{\mathbb {V}_{i}\}_{i=0}^{\infty }$ is convergent cauchy sequence in Hilbert space. $\square$

The uniqueness theory of solutions is one of important theorems in the qualitative analysis of models and has been mentioned in many references (see e.g.^24,56^). Therefore, we state it as follow:

#### Theorem 3

Let $\{\Psi _n\}$ is a sequence of solution such that $\Psi _n$ is converging at $n\rightarrow \infty$ then solution is unique.

#### Proof

Assume $\bar{\Psi }$ an approximate solution of the proposed problem and $\Psi _n$ is a general solution, then let $\Psi _{n}( z ,\tau )$ has two limits of convergence as $\Psi _n\rightarrow \bar{\Psi }$ and $\Psi _n\rightarrow \mathbb {U}$ as $n\rightarrow \infty$, then suppose that $\bar{\Psi }\ne \mathbb {U}$ and $\Arrowvert \bar{\Psi }-\mathbb {U}\Arrowvert = \varphi$. Then

16 $$\begin{aligned} \Arrowvert \bar{\Psi }-\mathbb {U}\Arrowvert =\Arrowvert \bar{\Psi }-\Psi _n+\Psi _n-\mathbb {U}\Arrowvert \\ \le \Arrowvert \bar{\Psi }-\Psi _n\Arrowvert +\Arrowvert \Psi _n-\mathbb {U}\Arrowvert , \end{aligned}$$

since

$\Psi _n\rightarrow \bar{\Psi }$, and $\Psi _n\rightarrow \mathbb {U}$, then $\Arrowvert \Psi _n\rightarrow \bar{\Psi }\Arrowvert \rightarrow 0$ and $\Arrowvert \Psi _n\rightarrow \mathbb {U}\Arrowvert$ as $n\rightarrow \infty$.

From Eq. (16), we obtain $\Arrowvert \bar{\Psi }-\mathbb {U}\Arrowvert \rightarrow 0$ as $n\rightarrow \infty$.

Hence the solution is unique. $\square$

#### Theorem 4

Assume that $\mathcal {D}^{i\zeta } \ \Psi (\tau ) \in \mathcal {C}[0,1],\ \forall \ i=0,1,...,n+1\ and\ 0<\zeta \le 1$. Let $\Psi _{n}(\tau )$ is the best square approximation of $\Psi (\tau )$, then :

$$\begin{aligned} \Arrowvert \Psi (\tau )-\Psi _n(\tau )\Arrowvert \le \dfrac{\mathscr {A} \mathscr {B}^{(n+1) \zeta }}{\Gamma (1+(n+1) \zeta )}, \end{aligned}$$

where $$\mathscr {A}=\max _{\tau \in [0,1]}\ \mathcal {D}^{(n+1)\zeta }\Psi (\tau )$$ and $$\mathscr {B}= \max \{\tau _0,\tau -\tau _0\}$$.

#### Proof

By using Taylor’s expansion for the function $\Psi (\tau )$ as:

$$\begin{aligned} \Psi (\tau )=\sum _{\Im =0}^n \dfrac{(\tau -\tau _0)^{\zeta \Im }}{\Gamma (1+\zeta \Im )}\mathcal {D}^{\zeta \Im }\ \Psi (\tau _0) +\dfrac{(\tau -\tau _0)^{(n+1) \zeta }}{\Gamma (1+(n+1) \zeta )}\mathcal {D}^{(n+1)\zeta }\ \Psi (\epsilon ),\quad \epsilon \in [\tau _0,\tau ], \forall \ \tau _0 \in [0,1]. \end{aligned}$$

Suppose

17 $$\begin{aligned} \tilde{\Psi }_{n}(\tau )=\sum _{\Im =0}^n \dfrac{(\tau -\tau _0)^{\zeta \Im }}{\Gamma (1+\zeta \Im )}\mathcal {D}^{\zeta \Im }\ \Psi (\tau _0), \end{aligned}$$

we have $$|{\Psi (\tau )- \tilde{\Psi }_{n}(\tau )}|=|{\dfrac{(\tau -\tau _0)^{(n+1) \zeta }}{\Gamma (1+(n+1) \zeta )}\mathcal {D}^{(n+1)\zeta } \Psi (\epsilon )}|.$$

Since, $\Psi _{n}(\tau )$ is the best square approximation for $\Psi (\tau )$, we get

$$\begin{aligned} \Arrowvert \Psi (\tau )-\Psi _n(\tau )\Arrowvert ^{2}\le \Arrowvert \Psi (\tau )- \tilde{\Psi }_{n}(\tau )\Arrowvert ^{2}\\ =\displaystyle \int _{0}^{1}\bigg (\Psi (\tau )- \tilde{\Psi }_{n}(\tau )\bigg )^{2}d\tau \\ \le \displaystyle \int _{0}^{1} \bigg ( \dfrac{(\tau -\tau _0)^{(n+1) \zeta } }{\Gamma \big (1+(n+1) \zeta \big )}\mathcal {D}^{(n+1)\zeta } \Psi (\epsilon )\bigg )^2 d\tau \\ =\mathscr {A}^{2}\displaystyle \int _{0}^{1} \bigg ( \dfrac{(\tau -\tau _0)^{(n+1) \zeta } }{\Gamma \big (1+(n+1) \zeta \big )}\bigg )^2 d\tau . \end{aligned}$$

Let $\mathscr {B}= \max \{\tau _0,\tau -\tau _0\}$, then we get

$$\begin{aligned} \Arrowvert \Psi (\tau )-\Psi _n(\tau )\Arrowvert ^{2}\le \bigg ( \dfrac{\mathscr {A}\mathscr {B}^{(n+1) \zeta } }{\Gamma \big (1+(n+1) \zeta \big )}\bigg )^2 \displaystyle \int _{0}^{1}d\tau \\ =\bigg (\dfrac{ \mathscr {A} \mathscr {B}^{(n+1) \zeta }}{\Gamma (1+(n+1) \zeta )}\bigg )^2. \end{aligned}$$

Then, by taking the square roots of both sides, we obtain

$$\begin{aligned} \Arrowvert \Psi (\tau )-\Psi _n(\tau )\Arrowvert \le \dfrac{\mathscr {A} \mathscr {B}^{(n+1) \zeta }}{\Gamma (1+(n+1) \zeta )}.\end{aligned}$$

$$\square$$

## Description of methodology 

In this section, we utilize confluent Bernoulli collocation approach for obtaining approximate solution of non-linear time-fractional KGP which defined in Eqs. (1) and (2), as following steps:

$\blacklozenge$ $\mathbf {Step \ I.}$ By substituting Eqs. (11) and (15) into Eq. (1), we have

18 $$\begin{aligned} {\left\{ \begin{array}{ll} \begin{aligned} \sum _{i=0}^n \mathfrak {D}^{\zeta }_{\tau } \lambda _{i}(\tau )\ \mathfrak {B}^{(a,b)}_i( z )+\rho \ \sum _{i=\lceil 2\rceil }^n \sum _{k=0}^{i-\lceil 2\rceil } \lambda _{i}(\tau )\ \mathcal {E}_{i,k} ^{(2)} z ^{i-k-2}\\ +\sigma \ \sum _{i=0}^n \lambda _{i}(\tau )\ \mathfrak {B}^{(a,b)}_i( z )+\varrho \bigg (\sum _{i=0}^n \lambda _{i}(\tau )\ \mathfrak {B}^{(a,b)}_i( z )\bigg )^{2}-\mathbb {H}( z ,\tau )=0. \end{aligned} \end{array}\right. } \end{aligned}$$

$\blacklozenge$ $\mathbf {Step \ II.}$ Now, we collocate Eq. (18) at $z _{r},$ $r = 0,1,2,...n-\lceil \zeta \rceil$ and the collocation point of confluent Bernoulli $\mathfrak {B}^{(a,b)}_{n+1-\lceil \zeta \rceil } ( z )$, we get system of fractional order differential equations (FODEs) as:

19 $$\begin{aligned} {\left\{ \begin{array}{ll} \begin{aligned} \sum _{i=0}^n \mathfrak {D}^{\zeta }_{\tau } \lambda _{i}(\tau )\ \mathfrak {B}^{(a,b)}_i( z _{r})+\rho \ \sum _{i=\lceil 2\rceil }^n \sum _{k=0}^{i-\lceil 2\rceil } \lambda _{i}(\tau )\ \mathcal {E}_{i,k} ^{(2)} z _r^{i-k-2}\\ +\sigma \ \sum _{i=0}^n \lambda _{i}(\tau )\ \mathfrak {B}^{(a,b)}_i( z _r)+\varrho \bigg (\sum _{i=0}^n \lambda _{i}(\tau )\ \mathfrak {B}^{(a,b)}_i( z _r)\bigg )^{2}-\mathbb {H}( z _{r},\tau )=0. \end{aligned} \end{array}\right. } \end{aligned}$$

$\blacklozenge$ $\mathbf {Step \ III.}$ By appling Eq. (11) into Eq. (2) at $z _{r}$, we obtain system of algebraic equations as:

20 $$\begin{aligned} {\left\{ \begin{array}{ll} \begin{aligned} \sum _{i=0}^n \lambda _{i}(0)\ \mathfrak {B}^{(a,b)}_i( z_r )=\mathfrak {F}_1 ( z_r ),\\ \sum _{i=0}^n \mathfrak {D}_{\tau }\lambda _{i}(0)\ \mathfrak {B}^{(a,b)}_i( z_r )=\mathfrak {F}_2 ( z_r ), \end{aligned} \end{array}\right. } \end{aligned}$$

where BCs

21 $$\begin{aligned} {\left\{ \begin{array}{ll} \begin{aligned} \sum _{i=0}^n \lambda _{i}(\tau )\ \mathfrak {B}^{(a,b)}_i(-1)=- \mathcal {W} (\tau ),\\ \sum _{i=0}^n \lambda _{i}(\tau )\ \mathfrak {B}^{(a,b)}_i(1)=\mathcal {W} (\tau ). \end{aligned} \end{array}\right. } \end{aligned}$$

To obtain the unknown coefficients $\lambda _i(\tau )$, $i=0,1,2,...,n$ combing Eqs. (19), (20) and (21), we have system of FODEs, which can be solved by utilizing RPSS.

To determine the unknown coefficients of $\lambda _i(\tau )$, we take $n=2$, $a=2,b=1$ in Eq. (19):

22 $$\begin{aligned} {\left\{ \begin{array}{ll} \mathcal {D}^{\zeta }_{\tau }\ \lambda _{0}(\tau )-\dfrac{7}{48}\mathcal {D}^{\zeta }_{\tau }\ \lambda _{2}(\tau )+6\ \rho \ \lambda _{2}(\tau )+\sigma \bigg ( \lambda _{0}(\tau )-\dfrac{7}{48} \lambda _{2}(\tau )\bigg )+\varrho \bigg (\lambda _{0}(\tau )-\dfrac{7}{48} \lambda _{2}(\tau )\bigg )^{2}-\mathbb {H}(\dfrac{1}{4},\tau )=0. \end{array}\right. } \end{aligned}$$

By solving Eq. (21), then we get

23 $$\begin{aligned} {\left\{ \begin{array}{ll} \begin{aligned} \lambda _{1}(\tau )= \dfrac{19}{32}\mathcal {W} (\tau )-\dfrac{3}{8}\lambda _{0}(\tau ),\\ \lambda _{2}(\tau )= \dfrac{3}{32}\mathcal {W} (\tau )-\dfrac{3}{8}\lambda _{0}(\tau ). \end{aligned} \end{array}\right. } \end{aligned}$$

By substituting Eq. (23) into Eq. (22), then

24 $$\begin{aligned} {\left\{ \begin{array}{ll} \begin{aligned} \dfrac{135}{128}\mathcal {D}^{\zeta }_{\tau }\ \lambda _{0}(\tau )-\dfrac{7}{512}\mathcal {D}^{\zeta }_{\tau }\ \mathcal {W}(\tau )+\ \dfrac{9\ \rho }{16} \ \mathcal {W} (\tau )-\dfrac{9\ \rho }{4} \ \lambda _{0}(\tau )\\ +\sigma \bigg ( \dfrac{135}{128}\lambda _{0}(\tau )-\dfrac{7}{512} \mathcal {W} (\tau )\bigg )+\varrho \bigg (\dfrac{135}{128}\lambda _{0}(\tau )-\dfrac{7}{512} \mathcal {W} (\tau )\bigg )^2-\mathbb {H}(\dfrac{1}{4},\tau )=0, \end{aligned} \end{array}\right. } \end{aligned}$$

25 $$\begin{aligned} {\left\{ \begin{array}{ll} \begin{aligned} \mathcal {D}^{\zeta }_{\tau }\ \lambda _{0}(\tau )-\dfrac{7}{540}\mathcal {D}^{\zeta }_{\tau }\ \mathcal {W}(\tau )+ \dfrac{8\ \rho }{15} \ \mathcal {W} (\tau )-\dfrac{32 \ \rho }{15} \ \lambda _{0}(\tau )+\sigma \bigg ( \lambda _{0}(\tau )-\dfrac{7}{540} \mathcal {W} (\tau )\bigg )\\ +\varrho \bigg (\dfrac{135}{128}\ \lambda _{0}^{2}(\tau )-\dfrac{7}{256} \mathcal {W}(\tau )\ \lambda _{0}(\tau ) +\dfrac{49}{276480}\ \mathcal {W}^{2}(\tau )\bigg )-\dfrac{128}{135}\mathbb {H}(\dfrac{1}{4},\tau )=0. \end{aligned} \end{array}\right. } \end{aligned}$$

RPSS assumes the solution of Eq. (25) using FPS at $\tau _0=0$ as:

26 $$\begin{aligned} \lambda _{0}(\tau )=\Omega _{0}+\Omega _{1}\tau +\sum _{h=1}^\infty \sum _{j=0}^l \mathbb {P}_{hj}\dfrac{\tau ^{h\zeta +j}}{\Gamma (h\zeta +j+1)}. \end{aligned}$$

Next, let $\lambda _{0(\kappa ,l)}(\tau )$ indicate the $\kappa ^{th}$ truncated series of $\lambda _{0}(\tau )$ which take the form:

27 $$\begin{aligned} \lambda _{0(\kappa ,l)}(\tau )=\Omega _{0}+\Omega _{1}\tau +\sum _{h=1}^\kappa \sum _{j=0}^l \mathbb {P}_{hj}\dfrac{t^{h\zeta +j}}{\Gamma (h\zeta +j+1)},\ \forall \kappa =1,2,... \ and\ l=0,1, \end{aligned}$$

where $\Omega _{0}$ and $\Omega _{1}$ can be obtained by solving Eqs. (20) and (21). The RPSS assumes the solution of Eq. (25) using FPS at $\tau _0=0$ as:

28 $$\begin{aligned} {\left\{ \begin{array}{ll} \begin{aligned} \mathbb {RES}_{(\kappa ,l)}(\tau )= \mathcal {D}^{\zeta }_{\tau }\ \lambda _{0(\kappa ,l)}(\tau )-\dfrac{7}{540}\mathcal {D}^{\zeta }_{\tau }\ \mathcal {W}(\tau )+ \dfrac{8\ \rho }{15} \mathcal {W} (\tau )-\dfrac{32\ \rho }{15} \lambda _{0(\kappa ,l)}(\tau )\\ +\sigma \bigg ( \lambda _{0(\kappa ,l)}(\tau )-\dfrac{7}{540} \mathcal {W} (\tau )\bigg )+ \dfrac{135\ \varrho }{128}\ \lambda _{0(\kappa ,l)}^{2}(\tau )-\dfrac{7\ \varrho }{256} \mathcal {W}(\tau )\ \lambda _{0(\kappa ,l)}(\tau )\\ +\dfrac{49\ \varrho }{276480}\ \mathcal {W}^{2}(\tau )-\dfrac{128}{135}\mathbb {H}(\dfrac{1}{4},\tau ), \end{aligned} \end{array}\right. } \end{aligned}$$

*and*

29 $$\begin{aligned} {\left\{ \begin{array}{ll} \begin{aligned} \mathcal {D}^{(h-1)\zeta }_{\tau }\mathcal {D}^{j}_{\tau }\ \mathbb {RES}_{(\kappa ,l)}(\tau _0)=0,\\ \forall \ h=1,2,...,\kappa \ and\ j=0,1,...,l. \end{aligned} \end{array}\right. } \end{aligned}$$

##  The proposed problems and norm errors

To investigate the accuracy and applicability of CBCA-RPSS, we presented numerical outcomes through an error norms $\mathscr {L}_{\infty }$, $\mathscr {L}_{2}$ and root mean square error (RMSE) which defined in^57^ as follows:

$$\begin{aligned} \mathscr {L}_{\infty }=\underset{0\le \iota \le \texttt{M}}{\max (\texttt{E}_\iota )},\\ \mathscr {L}_{2}=\sqrt{ \textbf{h}\displaystyle \sum _{\iota =0}^{\texttt{M}} \texttt{E}_\iota },\\ and \ RMSE =\sqrt{\displaystyle \sum _{\iota =0}^{\texttt{M}}\dfrac{\texttt{E}_\iota ^2}{\texttt{M}}}, \end{aligned}$$

where $$\texttt{E}_\iota =|{ \Psi _{\iota }(Exact\ solution)-\Psi _{\iota }(Approximate \ solution)}|$$.

**Problem 1.** Consider non-linear time-fractional KGP^44,45^

30 $$\begin{aligned} \mathcal {D}^{\zeta }_{\tau }\ \Psi ( z ,\tau )- \mathcal {D}^{2}_{ z }\ \Psi ( z ,\tau )+\Psi ^{2}( z ,\tau )=- z cos(\tau )+ z ^{2}cos^{2}(\tau ), \ 1<\zeta \le 2,\ z \in [-1,1],\ \tau >0, \end{aligned}$$

with to ICs and BCs:

31 $$\begin{aligned} {\left\{ \begin{array}{ll} \Psi ({z},0)={z}, & \mathcal {D}_{\tau } \Psi ({z},0)=0,\\ \Psi (-1,\tau )=-cos(\tau ), & \Psi (1,\tau )=cos(\tau ). \end{array}\right. } \end{aligned}$$

which has an exact solution at $\zeta =2$ as $\Psi ( z ,\tau )= z cos(\tau ).$

According to an explanation of the proposed method in “Description of methodology” section, then the approximate solution is:

$$\Psi _{2}( z ,\tau ) = \lambda _{0}(\tau )+ (2 z -\dfrac{1}{2})\lambda _{1}(\tau ) + (3 z ^2-2 z +\dfrac{1}{6})\lambda _{2}(\tau ),$$

where $$\lambda _{0}(\tau )=\dfrac{1}{4}-\dfrac{1}{4}\dfrac{\tau ^{\zeta }}{\Gamma (1+\zeta )}+\dfrac{1}{4}\dfrac{\tau ^{2\zeta }}{\Gamma (1+2\zeta )}-...,$$

$$\lambda _{1}(\tau )=\dfrac{19}{32}cos(\tau )-\dfrac{3}{8}\lambda _{0}(\tau )$$

and

$$\lambda _{2}(\tau )=\dfrac{3}{32}cos(\tau )-\dfrac{3}{8}\lambda _{0}(\tau ).$$

**Problem 2.** Consider non-linear time-fractional KGP^45^

32 $$\begin{aligned} \mathcal {D}^{\zeta }_{\tau }\ \Psi ( z ,\tau )- \mathcal {D}^{2}_{ z }\ \Psi ( z ,\tau )+\dfrac{\pi ^2}{4}\Psi ( z ,\tau )+\Psi ^{2}( z ,\tau )= z ^{2}sin^{2}(\dfrac{\pi \tau }{2}), \ 1<\zeta \le 2,\ z \in [-1,1],\ \tau >0, \end{aligned}$$

with to ICs and BCs:

33 $$\begin{aligned} {\left\{ \begin{array}{ll} \Psi ( z ,0)=0, & \mathcal {D}_{\tau } \Psi ( z ,0)=\dfrac{\pi \tau }{2},\\ \Psi (-1,\tau )=-sin(\dfrac{\pi \tau }{2}), & \Psi (1,\tau )=sin(\dfrac{\pi \tau }{2}). \end{array}\right. } \end{aligned}$$

which has an exact solution at $\zeta =2$ as $\Psi ( z ,\tau )= z sin(\dfrac{\pi \tau }{2}).$

By appling the proposed method, then we can obtain the approximate solution as: $\Psi _{2}( z ,\tau ) = \lambda _{0}(\tau )+ (2 z -\dfrac{1}{2})\lambda _{1}(\tau ) + (3 z ^2-2 z +\dfrac{1}{6})\lambda _{2}(\tau ),$

where $$\lambda _{0}(\tau )=\dfrac{\pi }{8}\tau -\dfrac{\pi ^{3}}{32}\dfrac{\tau ^{\zeta +1}}{\Gamma (2+\zeta )}+\dfrac{\pi ^{5}}{128}\dfrac{\tau ^{2\zeta +1}}{\Gamma (2+2\zeta )}-...$$,

$$\lambda _{1}(\tau )=\dfrac{19}{32}sin(\dfrac{\pi }{2}\tau )-\dfrac{3}{8}\lambda _{0}(\tau )$$

and 

$$\lambda _{2}(\tau )=\dfrac{3}{32}sin(\dfrac{\pi }{2}\tau )-\dfrac{3}{8}\lambda _{0}(\tau ).$$

**Problem 3.** Consider the non-linear equation^45^

34 $$\begin{aligned} \mathcal {D}^{\zeta }_{\tau }\ \Psi ( z ,\tau )+ \Psi ( z ,\tau )\mathcal {D}_{ z }\ \Psi ( z ,\tau )-6\mathcal {D}^{2}_{ z }\ \Psi ( z ,\tau )=2 z \tau cos(\tau ^{2})+ z sin^{2}(\tau ^{2}), \ 0<\zeta \le 1,\ z \in [0,1],\ \tau >0, \end{aligned}$$

with to ICs and BCs:

35 $$\begin{aligned} {\left\{ \begin{array}{ll} \Psi ( z ,0)=0,\\ \Psi (0,\tau )=0, & \Psi (1,\tau )=sin(\tau ^{2}). \end{array}\right. } \end{aligned}$$

which has an exact solution at $\zeta =1$ as $\Psi ( z ,\tau )= z sin(\tau ^{2}).$ According to the proposed method, then we can write the approximate solution as:

$$\Psi _{2}( z ,\tau ) = \lambda _{0}(\tau )+ (2 z -\dfrac{1}{2})\lambda _{1}(\tau ) + (3 z ^2-2 z +\dfrac{1}{6})\lambda _{2}(\tau ),$$

where $$\lambda _{0}(\tau )=\dfrac{1}{2}\dfrac{\tau ^{2\zeta }}{\Gamma (1+2\zeta )}-30 \dfrac{\tau ^{6\zeta }}{\Gamma (1+6\zeta )}+7560\dfrac{\tau ^{10\zeta }}{\Gamma (1+10\zeta )}-...$$,

$$\lambda _{1}(\tau )=\dfrac{1}{5}sin(\tau ^{2})+\dfrac{6}{5}\lambda _{0}(\tau )$$

and   

$$\lambda _{2}(\tau )=\dfrac{3}{5}sin(\tau ^{2})-\dfrac{12}{5}\lambda _{0}(\tau ).$$

##  Results and discussion

In this section, we present an obtained numerical outcomes for all problems through tables and graphics in two and three dimensions.

**Fig.1:**
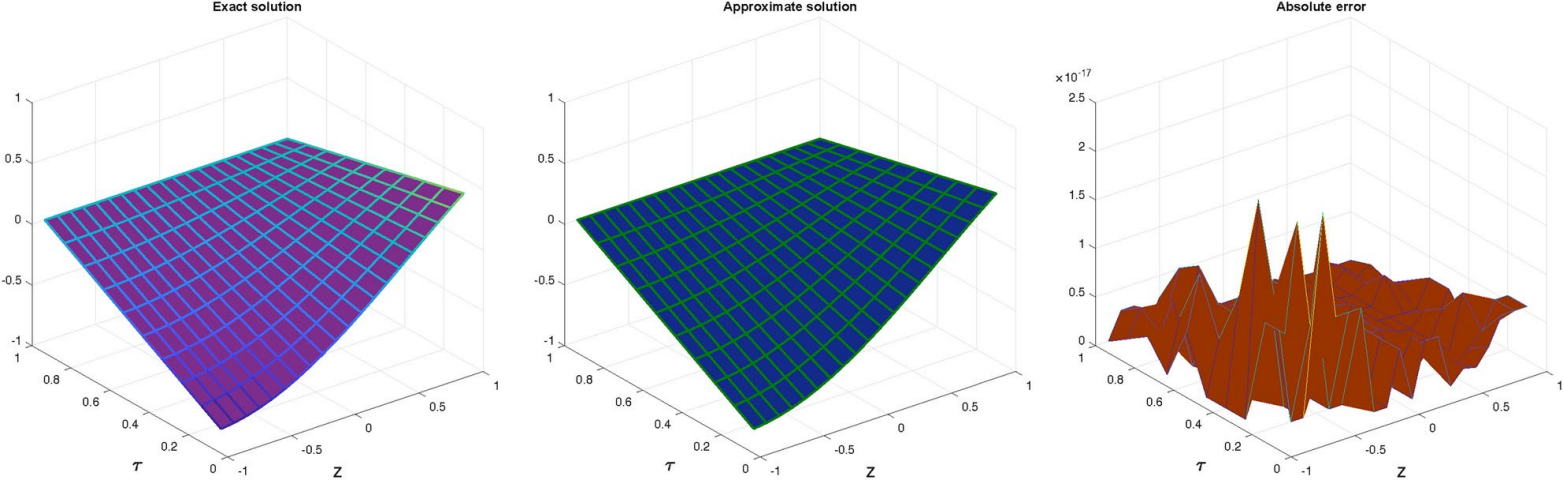
Graph of exact solution, CBCA-RPSS solution and absolute error at $\zeta =2$ for Problem 1.

**Fig. 2:**
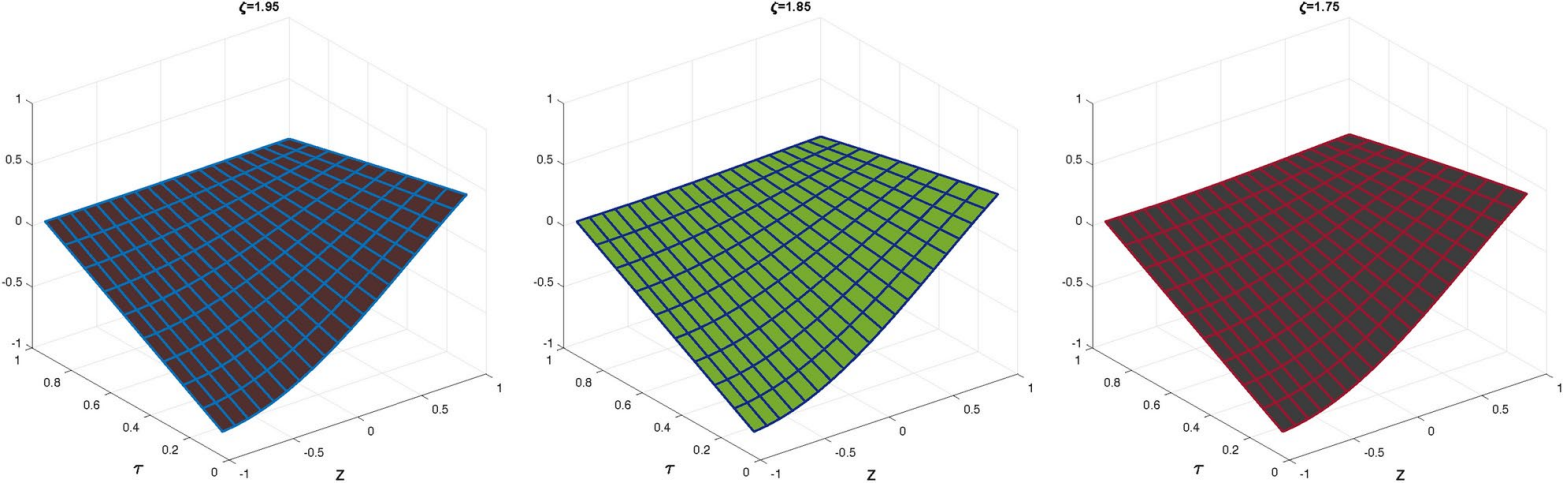
The CBCA-RPSS solution at various values of $\zeta$ for Problem 1.

**Fig. 3:**
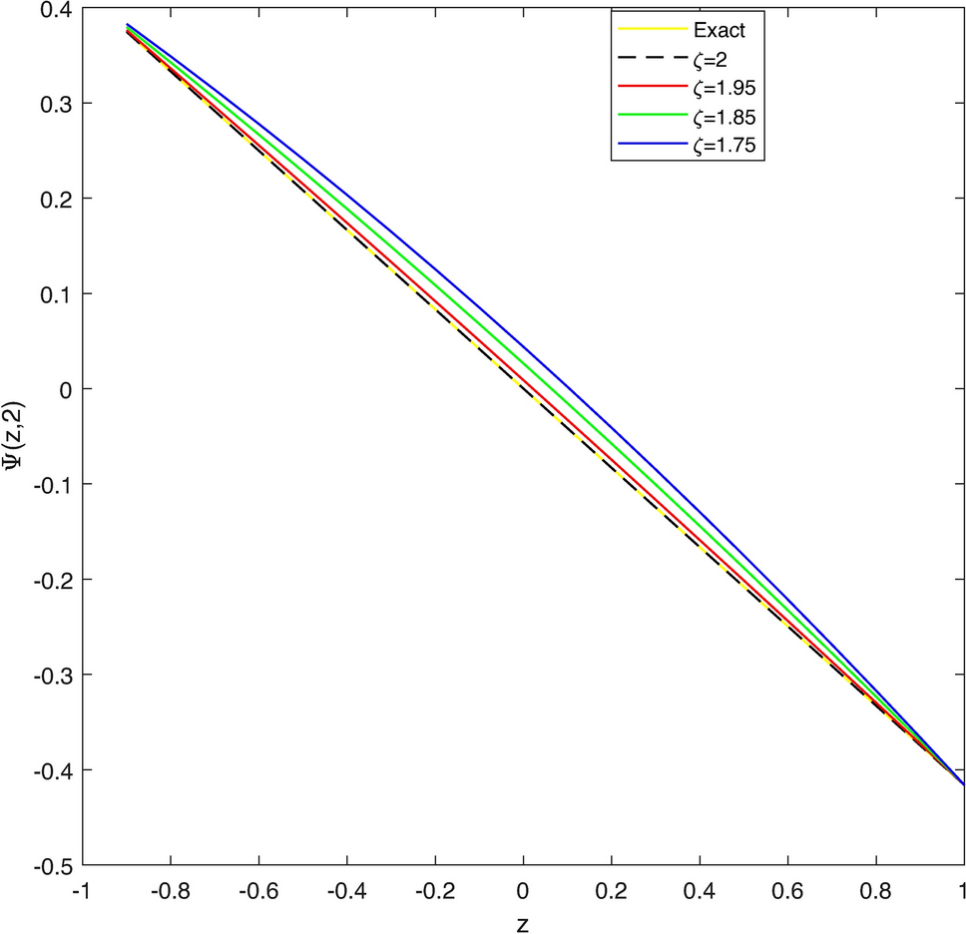
2D graph of exact and CBCA-RPSS solution with different values of $\zeta$ for Problem 1.

In Table 1, we compare the $\mathscr {L}_{\infty }$, $\mathscr {L}_{2}$ and RMS error norms obtained by CBCA-RPSS at $\zeta =2$ with Tension spline approach $O(k^2+k^2h^2+h^2)$ method^42^, $O(k^2+k^2h^2+h^4)$ method^42^, RBF approximation^43^, Clique polynomial method^44^ and BWCM^45^ of Problem 1. Figure 1 displays exact solution and CBCA-RPSS solution with its absolute error of Problem 1. Figure 2 expresses the comparison of CBCA-RPSS solutions for various fractional-order of $\zeta$ while Fig. 3 demonstrate the behavior of fractional-order $\zeta$ on CBCA-RPSS solution with exact solution at $\tau =2$ in 2D graph of Problem 1.

**Fig. 4:**
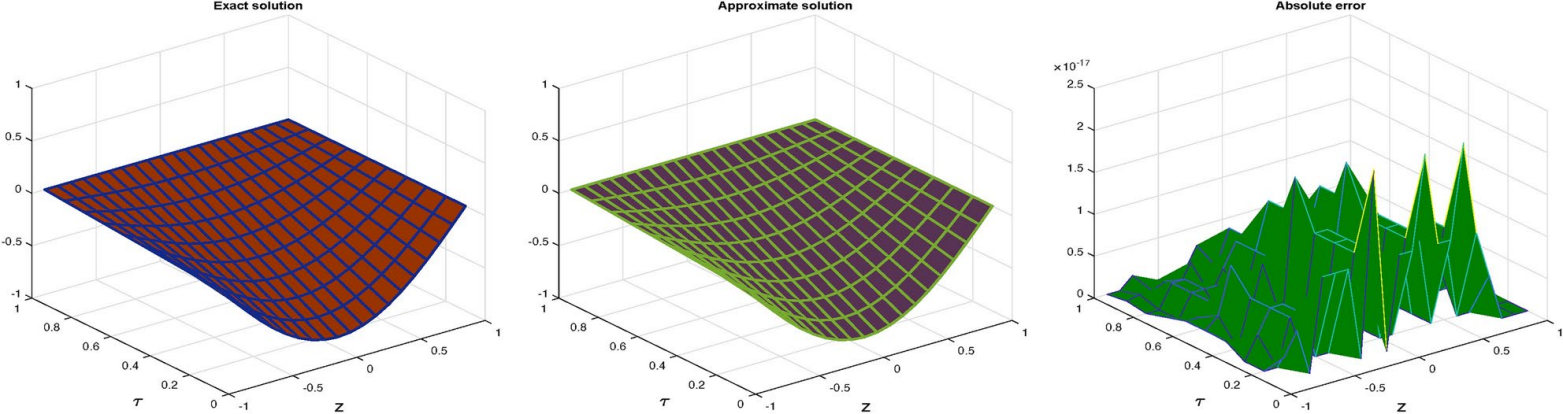
Graph of exact solution, CBCA-RPSS solution and absolute error at $\zeta =2$ for Problem 2.

**Fig. 5:**
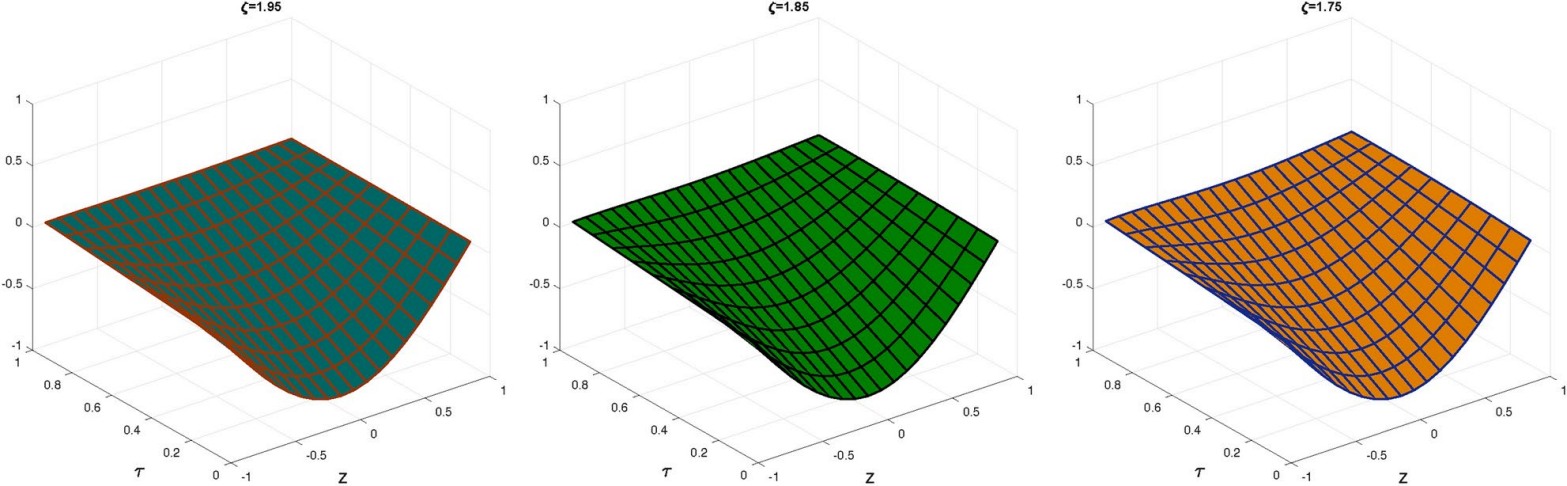
The CBCA-RPSS solution with various values of $\zeta$ for Problem 2.

**Fig. 6:**
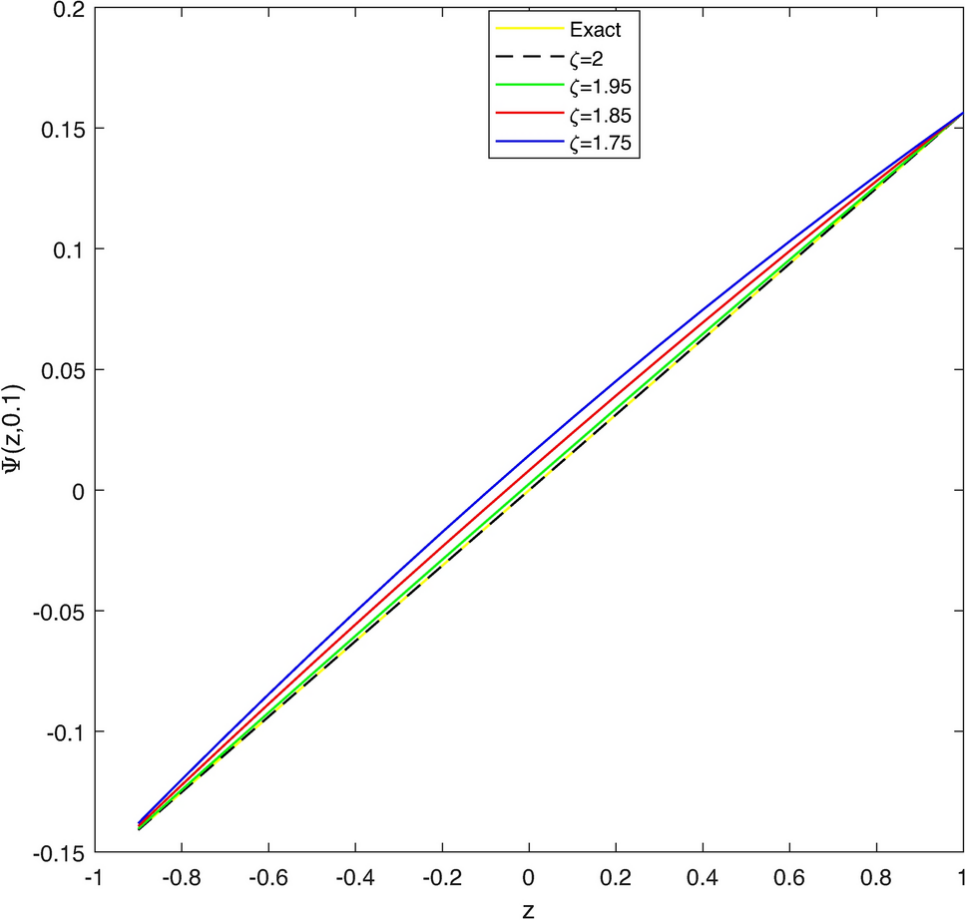
2D graph of exact and CBCA-RPSS solution with various values of $\zeta$ for Problem 2.

Table 2 display a comparison of error norms for the approximate solution by CBCA-RPSS at $\zeta =2$ with that Tension spline approach $O(k^2+k^2h^2+h^2)$ method^42^, $O(k^2+k^2h^2+h^4)$ method^42^, RBF approximation^43^ and BWCM^45^ of Problem 2. Figure 4 displays exact solution and CBCA-RPSS solution with its absolute error of Problem 2. Figure 5 expresses the comparison for CBCA-RPSS solution of various fractional-order of $\zeta$ while Fig. 6 show the behavior of fractional-order $\zeta$ on CBCA-RPSS solution with exact solution at $\tau =0.1$ in 2D graph of Problem 2.

**Fig. 7:**
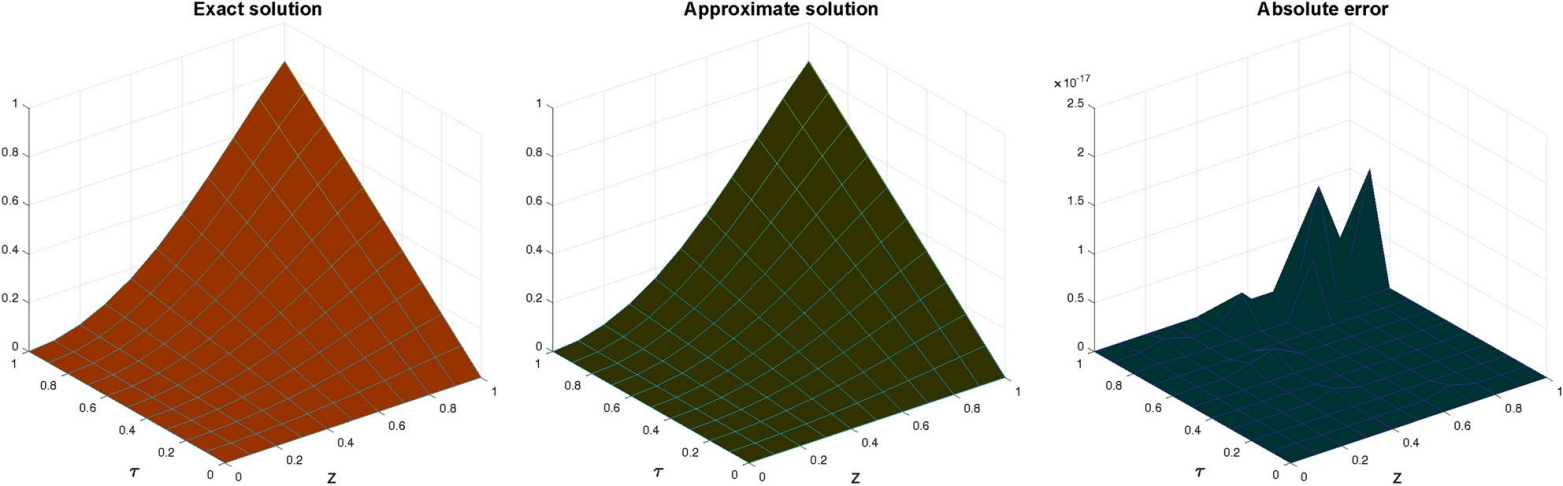
Graph of Exact solution, CBCA-RPSS solution and absolute error at $\zeta =1$ for Problem 3.

**Fig. 8:**
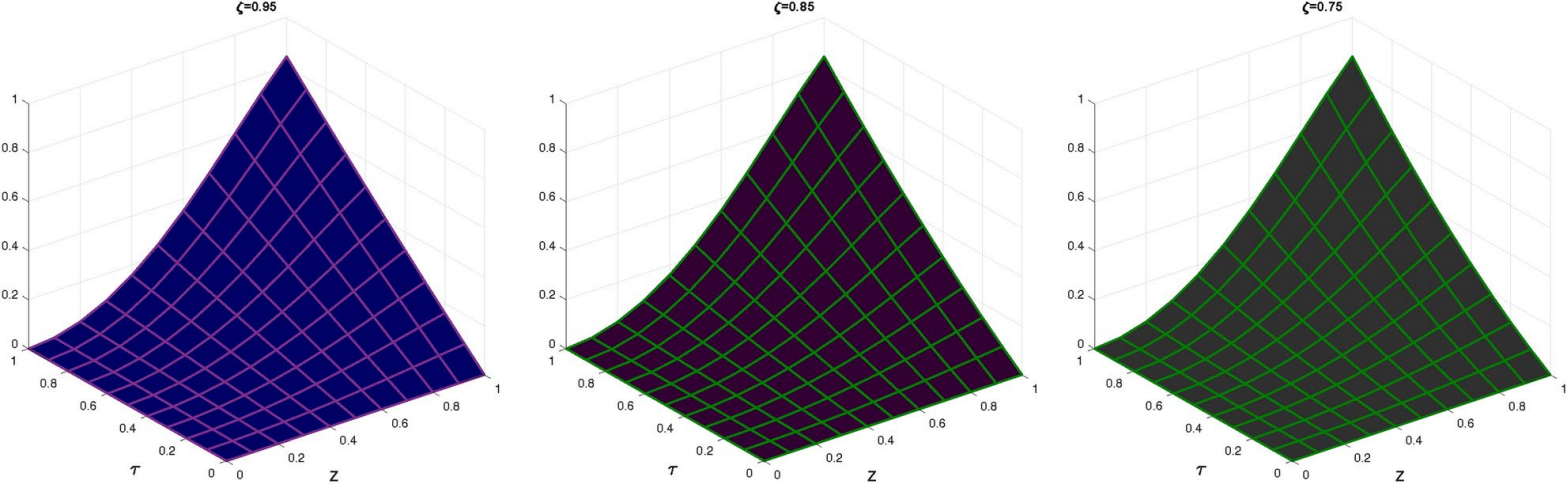
The CBCA-RPSS solution at different values of $\zeta$ for Problem 3.

**Fig. 9:**
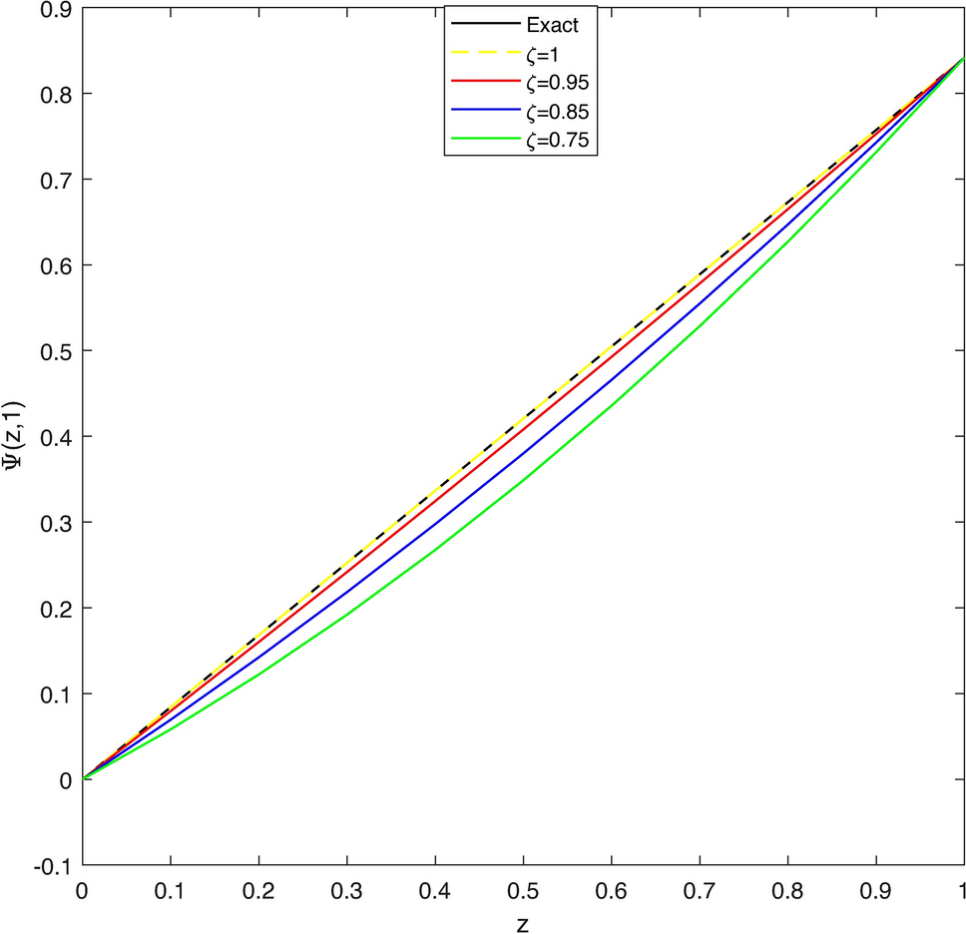
2D graph of Exact and CBCA-RPSS solution with various values of $\zeta$ for Problem 3.

In Table 3, the error norms are reported for $\zeta =1$ and compared with BWCM^45^ while in Table 4 show comparison of absolute error of CBCA-RPSS at $\zeta =1$ and $z =0.1$ with that BWCM^45^, ADM with the Bernstein polynomials (BPs)^58^ and ADM with modified Bernstein polynomials (MBPs)^58^ of Problem 3. Figure 7 displays exact solution and CBCA-RPSS solution with its absolute error of Problem 3. Figure 8 expresses the comparison for CBCA-RPSS solution of various fractional-order of $\zeta$ while Fig. 9 demonstrate the behavior of fractional-order $\zeta$ on CBCA-RPSS solution with exact solution at $\tau =1$ in 2D graph of Problem 3.

**Table 1:** Comparison of norm errors of CBCA-RPSS with other techniques at $\zeta =2$ and $z =0.1$ for problem 1.

$\tau$	$\mathscr {L}_{2}\ error$	$\mathscr {L}_{\infty }\ error$	RMSE
$O(k^2+k^2h^2+h^2)$ method^42^			
1	$7.01e{-}9$	$1.03e{-}9$	$6.97e{-}10$
3	$6.59e{-}9$	$1.00e{-}9$	$6.55e{-}10$
5	$1.29e{-}9$	$2.56e{-}10$	$1.28e{-}10$
7	$7.47e{-}9$	$1.13e{-}9$	$7.44e{-}10$
10	$5.84e{-}9$	$9.46e{-}10$	$5.81e{-}10$
$O(k^2+k^2h^2+h^4)$ method^42^			
1	$4.91e{-}9$	$7.68e{-}10$	$4.89e{-}10$
3	$4.69e{-}9$	$7.52e{-}10$	$4.66e{-}10$
5	$9.46e{-}10$	$1.76 e{-}10$	$9.41e{-}11$
7	$5.11e{-}9$	$7.63e{-}10$	$3.96e{-}10$
10	$3.98e{-}9$	$6.55e{-}10$	$5.81e{-}10$
RBF approximation^43^			
1	$6.54e{-}5$	$1.25e{-}5$	$6.50e{-}6$
3	$1.17e{-}4$	$1.55e{-}5$	$1.16e{-}6$
5	$2.20e{-}4$	$3.37e{-}5$	$2.19e{-}5$
7	$2.58e{-}4$	$3.77e{-}5$	$2.57e{-}5$
10	$7.98e{-}5$	$1.30e{-}5$	$7.94e{-}6$
Clique polynomial method^44^			
1	$3.98e{-}10$	$5.83e{-}12$	$3.86e{-}11$
3	$1.53e{-}10$	$7.35e{-}12$	$9.43e{-}11$
5	$6.34e{-}10$	$1.90e{-}11$	$3.97e{-}11$
7	$1.83e{-}10$	$4.73e{-}11$	$2.97e{-}11$
10	$5.34e{-}10$	$6.32e{-}11$	$5.37e{-}11$
BWCM^45^			
1	$8.16e{-}16$	$3.88e{-}16$	$2.46e{-}16$
3	$8.14e{-}15$	$4.21e{-}15$	$2.45e{-}15$
5	$6.68e{-}14$	$4.38e{-}14$	$2.61e{-}14$
7	$5.67e{-}13$	$2.86e{-}13$	$1.71e{-}13$
10	$4.08e{-}12$	$2.07e{-}12$	$1.23e{-}12$
CBCA-RPSS			
1	$1.55158e{-}18$	$6.93889e{-}18$	$3.46945e{-}19$
3	$3.10317e{-}18$	$1.38778e{-}17$	$6.93889e{-}19$
5	$0.00000e+00$	$0.00000e+00$	$0.00000e+00$
7	$0.00000e+00$	$0.00000e+00$	$0.00000e+00$
10	$0.00000e+00$	$0.00000e+00$	$0.00000e+00$

**Table 2:** Comparison of norm errors of CBCA-RPSS with other techniques at $\zeta =2$ and $z =0.5$ for problem 2.

$\tau$	$\mathscr {L}_{2}\ error$	$\mathscr {L}_{\infty }\ error$	RMSE
$O(k^2+k^2h^2+h^2)$ method^42^			
1	$2.71e{-}5$	$3.97e{-}6$	$2.69e{-}6$
2	$8.97e{-}6$	$1.51e{-}6$	$8.93e{-}7$
3	$1.49e{-}5$	$2.14e{-}6$	$1.48e{-}6$
4	$1.05e{-}5$	$1.86e{-}6$	$1.05e{-}6$
5	$3.36e{-}5$	$5.08e{-}6$	$3.34e{-}6$
$O(k^2+k^2h^2+h^4)$ method^42^			
1	$2.71e{-}5$	$3.97e{-}6$	$2.69e{-}6$
2	$8.97e{-}6$	$1.51e{-}6$	$8.93e{-}7$
3	$1.49e{-}5$	$2.14e{-}6$	$1.48e{-}6$
4	$1.05e{-}5$	$1.86e{-}6$	$1.05e{-}6$
5	$3.36e{-}5$	$5.08e{-}6$	$3.34e{-}6$
BWCM^45^			
1	$6.59e{-}16$	$3.33e{-}16$	$1.98e{-}16$
2	$1.18e{-}15$	$5.39e{-}16$	$3.58e{-}16$
3	$2.55e{-}14$	$1.25e{-}14$	$7.70e{-}15$
4	$1.27e{-}13$	$6.37e{-}14$	$3.85e{-}14$
5	$4.16e{-}13$	$2.08e{-}13$	$1.25e{-}13$
CBCA-RPSS			
1	$0.00000e+00$	$0.00000e+00$	$0.00000e+00$
2	$0.00000e+00$	$0.00000e+00$	$0.00000e+00$
3	$0.00000e+00$	$0.00000e+00$	$0.00000e+00$
4	$0.00000e+00$	$0.00000e+00$	$0.00000e+00$
5	$0.00000e+00$	$0.00000e+00$	$0.00000e+00$

**Table 3:** Comparison of norm errors of CBCA-RPSS with other techniques at $\zeta =1$ and $z =0.1$ for problem 3.

$\tau$	$\mathscr {L}_{2}\ error$	$\mathscr {L}_{\infty }\ error$	RMSE
BWCM^45^			
1	$2.6161e{-}12$	$1.0940e{-}12$	$7.8878e{-}13$
2	$8.1931e{-}10$	$3.9878e{-}10$	$2.4703e{-}10$
3	$7.4899e{-}9$	$3.7339e{-}9$	$2.2582e{-}9$
4	$3.0534e{-}8$	$1.5343e{-}8$	$9.2065e{-}9$
5	$8.5980e -8$	$4.3370e{-}8$	$2.5923e{-}8$
CBCA-RPSS			
1	$0.00000e+00$	$0.00000e+00$	$0.00000e+00$
2	$0.00000e+00$	$0.00000e+00$	$0.00000e+00$
3	$0.00000e +00$	$0.00000e+00$	$0.00000e+00$
4	$0.00000e+00$	$0.00000e+00$	$0.00000 \ e+00$
5	$1.93948e{-}19$	$1.73472e{-}18$	$8.26059e{-}20$

**Table 4:** Comparison of absolute error of CBCA-RPSS with other methods at $\zeta =1$ and $z =0.1$ for problem 3.

$\tau$	$\mathscr {A}_1$	$\mathscr {A}_2$	$\mathscr {A}_3$	$\mathscr {A}_4$
0	0.00000e+00	0.00000e+00	0.00000e+00	0.00000e+00
0.1	$5.5121e{-}6$	$4.1490e{-}6$	$1.4528e{-}16$	0.00000e+00
0.2	$3.4011e{-}5$	$1.8495e{-}6$	$3.9161e{-}15$	0.00000e+00
0.3	$8.8513 e{-}5$	$2.8381e{-}5$	$1.6276e{-}14$	0.00000e+00
0.4	$1.4044e{-}5$	$9.1215 e{-}5$	$3.8899e{-}14$	0.00000e+00
0.5	$1.2560 e{-}4$	$1.6204 e{-}4$	$7.0159e{-}14$	0.00000e+00
0.6	$4.2509 e{-}5$	$1.8804e{-}4$	$1.0514 e{-}13$	0.00000e+00
0.7	$4.3607e{-}4$	$1.0861e{-}4$	$1.3563e{-}13$	$6.93889e{-}18$
0.8	$1.0501 e{-}3$	$1.0202 e{-}4$	$1.5012e{-}13$	0.00000e+00
0.9	$1.7188e{-}3$	$3.7522e{-}4$	$1.3385e{-}13$	0.00000e+00
1	$2.0247e{-}3$	$4.8632e{-}4$	$6.8667e{-}14$	0.00000e+00

$\mathscr {A}_1$ absolute error by ADM with BPs^58^

$\mathscr {A}_2$ absolute error by

ADM with MBPs^58^

$\mathscr {A}_3$ absolute error by BWCM^45^

$\mathscr {A}_4$ absolute error by CBCA-RPSS

## Concluding remarks

   In this paper, CBCA-RPSS was successfully used to obtain an approximate solution for non-linear time-fractional KGP. First, by utilizing confluent Bernoulli polynomial and their properties the non-linear time-fractional KGP is reduced into a system of FODEs. Secondly, the RPSS is used to solve these system of equations with helping of the given initial and boundary conditions. We derived some theories of convergence, uniqueness and error analysis of proposed method. Finally, the comparisons of results abtained by CBCA-RPSS were performed and found that it is very computationally accurate than other existing techniques in literature^42–45,58^. Also, this comparisons shows that present method is very straightforward, simple, accurate and can be applied to solve nonlinear PDE problems that arise in engineering problems and complex phenomena.

## Data Availability

Data used to support the findings of this study are included in the article.
